# Mucus-Pathogen Interactions in the Gastrointestinal Tract of Farmed Animals

**DOI:** 10.3390/microorganisms6020055

**Published:** 2018-06-18

**Authors:** Macarena P. Quintana-Hayashi, Médea Padra, János Tamás Padra, John Benktander, Sara K. Lindén

**Affiliations:** Department of Medical Biochemistry and Cell biology, Institute of Biomedicine, Sahlgrenska Academy, University of Gothenburg, Box 440, 405 30 Gothenburg, Sweden; macarena.quintana.hayashi@gu.se (M.P.Q.-H.); medea.padra@gu.se (M.P.); janos.tamas.padra@gu.se (J.T.P.); john.benktander@gu.se (J.B.)

**Keywords:** mucus, mucin, *O*-glycosylation, host-pathogen interaction, stomach, intestine, bacteria, parasite, farm animal

## Abstract

Gastrointestinal infections cause significant challenges and economic losses in animal husbandry. As pathogens becoming resistant to antibiotics are a growing concern worldwide, alternative strategies to treat infections in farmed animals are necessary in order to decrease the risk to human health and increase animal health and productivity. Mucosal surfaces are the most common route used by pathogens to enter the body. The mucosal surface that lines the gastrointestinal tract is covered by a continuously secreted mucus layer that protects the epithelial surface. The mucus layer is the first barrier the pathogen must overcome for successful colonization, and is mainly composed of densely glycosylated proteins called mucins. The vast array of carbohydrate structures present on the mucins provide an important setting for host-pathogen interactions. This review summarizes the current knowledge on gastrointestinal mucins and their role during infections in farmed animals. We examine the interactions between mucins and animal pathogens, with a focus on how pathogenic bacteria can modify the mucin environment in the gut, and how this in turn affects pathogen adhesion and growth. Finally, we discuss analytical challenges and complexities of the mucus-based defense, as well as its potential to control infections in farmed animals.

## 1. Introduction

### 1.1. Animal Production and Infection Challenges

Gastrointestinal (GI) pathogens affect livestock, poultry, and aquaculture production systems worldwide, decreasing overall production parameters and causing considerable economic losses. Besides their effect in increased morbidity, mortality, and reduced feed conversion rates in food animals, GI pathogens can also decrease the safety of food products due to their zoonotic potential.

Intensive food animal production systems require the implementation of biosecurity, prophylactic, and therapeutic measures to prevent and control disease. High animal density in farms in conjunction with stress factors facilitate pathogen transmission. In aquaculture, for example, stocking density and stress are important factors that can increase susceptibility to infections such as those caused by *Aeromonas salmonicida* and *Vibrio* spp. [1,2]. Important bacterial pathogens in poultry include *Salmonella* spp., *Escherichia coli*, and *Clostridium* spp. [3,4,5]. *Clostridium perfringens* is a causative agent of necrotic enteritis (NE), a highly prevalent disease in poultry [6]. Additionally, parasitic coccidial infections by *Eimeria* spp. are common and affect the poultry industry [7]. Enteric infections due to *E. coli*, *C. perfringens*, *Brachyspira hyodysenteriae*, and *Lawsonia intracellularis* can reduce productivity and welfare in pigs [8]. *Helicobacter suis* infection in the pig stomach is associated with gastritis and decreased daily weight gain [9]. The GI parasites *Ostertagia ostertagi* and *Cooperia oncophora* have a significant impact in cattle production, mainly affecting the abomasum and intestines, respectively [10,11].

Antimicrobials are extensively used in intensive livestock, poultry, and aquaculture production for diverse purposes including therapeutic (i.e, treatment of diseases) and/or non-therapeutic purposes (i.e., disease control, prophylaxis and growth promotion) [12,13]. However, the use of antimicrobials in food animals has come under scrutiny due its association with the emergence of antimicrobial resistant strains, particularly those that can infect humans [14,15]. Studies have linked the use of antimicrobials for growth promotion, where they are continuously administered in sub-therapeutic doses via feed or water, and antimicrobial resistant bacteria of animal origin [16,17,18]. Pathogens becoming resistant to antimicrobials are a public health issue, and the concerns raised regarding the use of antimicrobials for non-therapeutic purposes in food animals has led to their complete ban in the European Union [19]. As a result, the incidence of several animal diseases such as NE in poultry and diarrhea due to *E*. *coli*, *L*. *intracellularis*, and *Brachyspira* spp. in pigs, which were previously under control by in-feed antimicrobials, has increased [20,21,22,23]. Resistance to antimicrobials used in the control and treatment of nematodes has been reported in livestock [24,25]. Consequently, alternative strategies to prevent and treat infections in farmed animals are needed to decrease the risk to human health and increase animal health and productivity.

### 1.2. Mucus, Mucins, and Mucin Glycosylation

GI mucus and mucins from primates and rodents have been previously reviewed elsewhere [26,27], therefore, we only present a brief summary as a general introduction to mucus in farmed animals.

#### 1.2.1. Mucus

The gastrointestinal mucosa continuously secretes mucus, which consists of 95% water and the remaining 5% is mainly composed of gel-forming highly glycosylated mucin glycoproteins, responsible for the viscous properties of mucus [28,29]. Mucus also contains salts, lipids, and proteins with protective functions, such as lysozyme, immunoglobulins, defensins, growth factors, and trefoil factors [30,31,32,33]. Mucins provide a matrix keeping other secreted defensive molecules in a strategic location [26,34]. Together these components create a slimy organized layer, which is the first barrier pathogens encounter [26,34]. Mucins form large netlike structures, which expand and replenish the mucus from underneath [26,34]. The thickness of the mucus layer is approximately 250 μm in the rat stomach, 150–400 μm in the rat small intestine, and reaching 800–900 μm in the rat colon [35]. Dietary fiber and microbiota have been demonstrated to be important for the generation of a mucus layer during normal conditions in mice [36,37].

#### 1.2.2. Mucins

Mucins are highly glycosylated glycoproteins that generally are large, with the secreted multimeric mucins having a molecular weight in the order of 40 MDa [38]. Mucins consist of a protein core with regions rich in serine and threonine, where *O*-linked carbohydrate chains are added in the Golgi apparatus during biosynthesis [39]. The two major types of mucins are transmembrane (cell-surface) and secretory mucins. Transmembrane mucins are located on the apical surface of mucosal epithelial cells, and they participate in mucosal defense translating external stimuli to cellular responses [40,41]. In the human gastrointestinal tract, cell-surface mucins include mucin-1 (MUC1), MUC3, MUC4, MUC12, MUC13, MUC15, MUC16, and MUC17 [42].

Secretory mucins can be gel-forming (MUC2, MUC5AC, MUC5B, MUC6, and MUC19) or non-gel-forming (MUC7). In the healthy human stomach, MUC5AC and MUC6 are the major gel-forming mucins located in the surface and glandular region, respectively [43], whereas in the intestine the main secreted mucin is MUC2 [44]. In the GI tract, secretory mucins are produced and secreted by mucous cells of glandular tissues and specialized epithelial cells called goblet cells [45].

#### 1.2.3. Mucin Glycosylation

Post-translational modifications of proteins by glycosylation can occur in *N*-linked and *O*-linked form, where *O*-glycosylation is the main modification of mucins. Mucin-type *O*-glycosylation takes place in the Golgi complex and is initiated by the addition of α-*N*-acetylgalactosamine (GalNAc) to the hydroxyl group of Ser/Thr side chains [46,47]. The complex oligosaccharides on proteins have three regions: core region (core 1–core 8), backbone region (type 1 and type 2), and peripheral region. This latter region can be terminated by fucose, galactose, GalNAc, or sialic acid residues, forming histo-blood-group antigens such as A, B, H, Lewis a (Le^a^), Lewis b (Le^b^), Lewis x (Le^x^), Lewis y (Le^y^), as well as sialyl-Le^a^ and sialyl-Le^x^ structures [48]. The glycans can be further modified by sulfation. The structure of carbohydrates depends on glycosyltransferases expressed by the cell: this is in turn governed by the inherited genetics (i.e., blood group antigens) as well as by tissue location [49]. The mucin oligosaccharide terminal structures vary between species, individuals, and tissue locations within each individual [50,51,52,53,54]. Mucin *O*-glycans contribute up to 80% of the molecular weight of mucins [28]. The high level of glycosylation enables mucins to function as a protective barrier by lubricating the epithelium and preventing degradation of the protein backbone by proteases [55,56]. Moreover, mucin glycans can modulate cell adhesion, serve as ligands for cell surface receptors, take part in host-pathogen interactions and serve as energy sources for both commensals and pathogens [57,58,59,60,61,62].

### 1.3. Host-Pathogen Interactions in the Mucus Niche

Microbes commonly interact with the glycan structures on the host’s glycocalyx to colonize mucosal surfaces [63]. The high variety of mucin oligosaccharides forms an extensive repertoire of attachment sites for microorganisms [54,64,65,66], which attach to mucosal glycans via adhesins with different carbohydrate specificities [67,68,69]. Both membrane bound and secreted mucins can play protective roles during infection. Mice lacking the membrane bound mucin Muc1 are more susceptible to *Helicobacter pylori* and *Campylobacter jejuni* infections [70,71]. MUC1 expression is upregulated in response to infection, and it also acts as a releasable decoy limiting adhesion of the human gastric pathogen *H. pylori* to the cell surface [40]. In humans, polymorphisms in MUC1 can contribute to the development of gastritis, gastric cancer, and Crohn’s disease [72,73]. 

The protective role of secreted intestinal mucins has also been demonstrated in mice lacking Muc2 with increased susceptibility to *Salmonella enterica* and *Citrobacter rodentium* infections [74,75], while Muc5C deficiency hinders *Trichuris muris* clearance [76]. Furthermore, *H. pylori*-infected rhesus monkeys and children secreting mucins with low *H. pylori* binding capacity develop higher *H. pylori* density infections and gastritis than individuals with mucins with high *H. pylori* binding capacity [53,65], suggesting that binding of pathogens to secreted mucins protects the epithelium. Potentially, binding to mucins hinders the pathogen from close contact with the epithelial cells, and aids in pathogen dissemination with gastric emptying. Adhesin dependent binding to mucin glycans can also cause aggregations that inhibit bacterial proliferation, and affect the expression of pathogen virulence genes [57,77].

Bacteria can also utilize mucus to their advantage: *H. pylori* is mainly located deep in the mucus layer, which protects the bacterium from gastric acid [78]. Mucus provides nutrients for bacterial growth [79,80]. A number of bacterial strains are able to degrade mucins by producing specific bacterial enzymes, including glycosidases, and use the released glycans as an energy source [81,82,83,84,85,86]. Studies comparing germ free and monoassociated mice have revealed that *Bacteroides thetaiotaomicron* induces the expression of fucose on cell surface glycoconjugates by secreting signaling molecules, and these fucosylated glycans can be utilized by these bacteria as a carbon source [87].

Host mucin glycans can regulate the proliferation and gene expression of pathogens [77,88,89]. In vitro proliferation assays revealed that culturing *H. pylori* in the presence of purified human gastric mucins can have a stimulating or an inhibitory effect on bacterial growth depending on the mucin type [57,77]. The expression of genes important for *H. pylori* pathogenicity can also be differentially regulated by mucins [77]. Similarly, *C. jejuni* exposure to MUC2 upregulates genes involved in pathogenicity and colonization [90].

Bacterial colonization can alter mucin production and glycosylation quantitatively and qualitatively [50]. For example, *H*. *pylori* infection can alter the expression of normal gastric mucins [91]. Furthermore, murine infection studies revealed that *H. pylori* impairs mucin production and turnover rate in the gastric mucosa [92]. *H. pylori* can express the blood-group binding adhesin BabA and/or the Sialic acid binding adhesin SabA, which bind to Le^b^ and sialylated Lewis a (SLe^a^) and SLe^x^ antigens on gastric mucins and glycolipids [61,69]. Primate infection with this pathogen caused time-dependent suppression of fucosylated blood group antigens, decreasing Le^b^ expression, while the expression of SLe^a^ and SLe^x^ antigens increased in the gastric mucosa, affecting the ability of the bacterium to adhere to the host epithelium [65].

## 2. Pig

### 2.1. Pig Gastric Mucins

Pigs are monogastric animals with a gastric mucosa that can be divided into two main parts: a glandular part (containing cardiac gland zone, fundic gland zone, and antrum with pyloric glands) and a nonglandular part called *pars esophagea* that is covered by a stratified squamous epithelium [93]. The nonglandular region and the cardiac gland zone have a pH between 5 and 7 due to the presence of saliva and cardiac gland bicarbonate secretions [94], while the fundic glands provide a lower pH in the distal part of the stomach [95]. The glandular part of the stomach produces mucins, and the apoprotein content and length of the glycosylated domains differ between mucins isolated from the surface epithelium and the glands, which may represent the pig equivalents of human Muc5ac and Muc6 mucins, respectively [96]. Proteomic analysis of pig gastric mucins identified mainly pig Muc5ac, but also indicated that Muc6 and Muc5b may be part of the mucin repertoire in the healthy pig stomach [97]. Similar to the human tissue localization, pig Muc5ac also localized to the surface epithelium [97]. For an overview on pig gastric mucins and glycosylation refer to Table 1.

The porcine mucins show inter-individual differences regarding density and glycan profile [97]. Mass spectrometric analysis identified 109 *O*-glycan structures in purified gastric mucins from three healthy pigs, out of which only 14 were present in all examined samples [97]. Similar to human gastric mucins, the oligosaccharides in pig mucins are mainly extended core 1 and core 2 glycans, but extended core 3 and core 4 structures are also present [97,98]. Pig gastric mucin glycans are mainly terminated by galactose and have a low degree of sialylation. Unlike human gastric mucins from healthy individuals [64], pig gastric mucins are highly sulphated [97,99].

The high acidity of the pig stomach provides a challenging environment for bacteria, thus relatively low numbers of acid-tolerant bacteria colonize this part of the gut [110,111]. Some of these microbes have been described to have beneficial functions for the host [112,113]. For example, lactic acid bacteria, such as *Lactobacillus* spp., can prevent the colonization of the gut by pathogens and enhance mucosal immunity [114,115,116]. Another advantageous function of some gut bacteria is the contribution to the availability of nutrients that can be used by the host [117]. Sequencing analysis of bacterial DNA revealed the presence of *Herbiconiux* and *Brevundimonas* genera in the pig stomach [111], which take part in the degradation of plant-derived food [118,119]. Spiral-shaped bacteria frequently colonize the pig stomach [120,121,122], and their presence has been associated with the development of chronic gastritis and decreased daily weight gain in pigs [9,123,124]. The main *Helicobacter* species in the pig stomach is *H. suis* [93], colonizing 60–95% of pigs at slaughter [125]. Fluorescence in situ hybridization performed on gastric tissue sections from *H. suis* infected pigs demonstrated that it colonizes the mucus layer lining the surface epithelium and the gastric pits of the pig stomach [97]. *H. suis* can be often found in close contact with parietal cells affecting acid production [97,126,127,128]. *H. suis* adhesion to glycans present in the pig stomach occurs via two modes: to Galβ3GlcNAcβ3Galβ4Glcβ1 at both neutral and acidic pH, and to negatively charged structures at acidic pH [97]. Furthermore, binding occurs to both mucins and glycolipids (Table 2) [97]. Binding to glycolipids confers close adherence to host epithelial cells, whereas mucins can act as decoys and hinder access to the epithelial cell surface, thus the balance between adhesion to mucins versus glycolipids is probably important for the colonization process and the outcome of the disease.

### 2.2. Pig Intestinal Mucins

The colonic mucus layer in healthy pigs, as in mice and humans, is organized in striations parallel to the mucosal surface and is mainly composed of Muc2 [44,101,141,142]. Glucosamine, galactosamine, galactose, fucose, and sialic acid are the main carbohydrates found on mucin glycoproteins in the pig colon [52,143]. Lectin staining of sections from small intestine suggests that the blood group AO genotype affects the glycomic profile of the mucosa [144]. A detailed characterization of the mucin glycosylation profile in the colon of healthy pigs identified high inter-individual variation of *O*-glycan structures, and equal distribution of *N*-acetylneuraminic acid (NeuAc) and *N*-glycolylneuraminic acid (NeuGc) containing structures [52]. Mucin-type cores 1, 2, 3, and 4 where detected with a predominance of core 4 glycans (Table 1) [52].

Pathogen-mucin interaction studies in the pig colon have shown that *S. enterica*, *Brachyspira hampsonii*, *B*. *hyodysenteriae*, and *Trichuris suis* increased the levels of Muc5ac (Table 2) [100,101,132,133]. The host’s response to the colonic pathogen *B*. *hyodysenteriae*, commonly associated with swine dysentery (SD), has been thoroughly studied from a mucin perspective. *B. hyodysenteriae* infection results in profound structural changes to the mucus layer, massively inducing mucus production with increased expression of Muc2 and de novo synthesis of Muc5ac [100,101]. Further studies determined that neutrophil elastase and interleukin (IL) 17 expressed in the pig colon during infection synergistically with *B*. *hyodysenteriae* induced mucin production via mitogen-activated protein kinase 3 [145]. Glycosylation changes in intestinal mucins can occur as an adaptation to new dietary constituents (i.e., during weaning), physical environment, as well as commensal and pathogenic bacteria [146,147,148,149,150,151,152]. GI bacterial pathogens can induce mucosal glycosylation changes, which in turn can affect bacterial growth and adhesion [57,65,77]. Challenges with the intestinal nematode *Trichinella spiralis* increased the amount of mucin in goblet cells, and caused changes in sulfation and sialylation in pigs, as determined by histochemical staining [134]. *B. hyodysenteriae* can also regulate mucin glycosylation synthesis in the pig colon [52]. Structural analysis of the mucins induced during *B*. *hyodysenteriae* infection compared to healthy controls showed loss of interindividual variation, shorter glycan chains, and higher abundance of neutral, core 2 and NeuGc containing structures [52]. Additionally, lower abundance of fucosylated and sulfated *O*-glycans was observed [52]. The increased abundance of shorter mucin *O*-glycans suggests a faster mucin turnover due to the increased mucin secretion during infection [52]. Furthermore, the mRNA levels of *C1GalT*, *C3GnT*, and *C2/4GnT-M* glycosyltranferases were upregulated in the colon tissue of *B*. *hyodysenteriae* infected pigs, linking differences in core glycan abundance to enzyme levels.

Motility and chemotaxis are important virulence factors that can facilitate intestinal colonization. *B. hyodysenteriae* has chemotactic behavior towards mucin, fucose, and serine [153,154,155]. Studies on *E*. *coli* and *H*. *pylori* indicate that adhesion to mucins favors the host by limiting colonization and access to the epithelial surface [70,156,157,158]. *E*. *coli* uses fimbrial adhesion factors to bind to the mucus layer and colonize the small intestine [129,130,131]. Meijerink et al. showed that a DNA polymorphism influences an alpha(1,2)fucosyltransferase activity related to blood group variants, affecting the mucin phenotype and thus the adhesion of enterotoxigenic *E*. *coli* F18 [159].

*E. coli* K99 fimbriae has affinity for sialic acid residues on mucin glycopeptides and glycolipids found in the pig small intestine [129,130,131]. *B. hyodysenteriae* also binds to mucin carbohydrates in the mucus layer, with increased adhesion ability to mucins from infected pigs [101]. However, it is yet to be determined the specific glycan structures that *B*. *hyodysenteriae* interacts with and whether the binding to colonic mucins is beneficial for the host or the pathogen. Studies have shown that pig intestinal bacteria can use mucin glycoproteins or its components as a carbon source [160,161,162]. *Clostridium* and *Bacteroides* isolated from pig colon mucosa are able to hydrolyze pig colonic and gastric mucin carbohydrates [160]. Similarly, several bacterial isolates belonging to the order *Clostridiales* produced butyrate and grew on mucin [161]. Recently, a novel commensal mucin degrading and butyrate producing bacterium, *Cloacibacillus porcorum*, was isolated from the pig intestinal mucosa [162].

## 3. Chicken

Avian species are monogastric animals with a glandular stomach termed proventriculus that secretes hydrochloric acid (HCl) and pepsinogen. Unlike non-avian animals, chickens have in addition a gizzard to breakdown food, a crop for food storage, two ceca pouches, and a cloaca that serves as a common cavity and opening for the GI, urinary and reproductive tracts [163].

Lang et al. [164] described that *Muc4*, *Muc13*, and *Muc16* transmembrane mucins, and *Muc6*, *Muc2*, *Muc5ac*, and *Muc5b* gel forming mucins are encoded in the chicken genome and are homologous to the human counterparts. Forder et al. [102] found detectable levels of *Muc2* and *Muc13*, but not *Muc1*, *Muc4*, *Muc5b*, and *Muc16* mRNA in the jejunum of healthy chickens (Table 1). The *Muc2* expression increases from anterior to posterior through the GI tract, and the *Muc2* proline, threonine, and serine (PTS) rich domain diverges from the human motif in amino acid composition and chain length [165].

Struwe et al. [103] characterized the mucin glycosylation profile in healthy chicken caecum, small intestine, and large intestine, detecting predominance of core 3 and core 4 *O*-glycans in all sections, and an increasing glycan diversity from the caecum towards the small and large intestine. Similarities in the distribution of neutral, sialylated, and sulfated *O*-glycans were observed in the small and large intestine (Table 1) [103]. The large intestine is the only site with NeuAc/NeuGc containing structures, whereas fucosylated mucin carbohydrates are found throughout the chicken small and large intestine as blood group H, B, and Lewis^a/x^ like structures [103]. 

The host’s defense against infection can result in differences in mucin expression, as is the case in necrotic enteritis infection in the small intestinal mucosa. Broiler chickens co-challenged with the coccidian parasite *Eimeria* spp. and *C*. *perfringens* resulted in a 54% increase in *Muc5ac* mRNA levels compared to unchallenged controls, while *Muc2* and *Muc13* expression levels decreased (Table 1) [102]. Furthermore, inoculation with *Eimeria* spp. increases mucus secretion, which may be the reason for increased growth and colonization of the mucolytic *C*. *perfringens* in the intestine of *Eimeria* spp. infected chicks [166]. *Eimeria tenella* binds to mucins isolated from the duodenum, which inhibits epithelial cell invasion in vitro [167]. Several studies related to bacterial interactions with mucins in chickens refer to *C*. *jejuni* colonization. Although *C*. *jejuni* is not pathogenic in chickens, it is an important enteric pathogen in humans, and chickens are the main source of infection. In chickens, *C*. *jejuni* is localized in the mucus layer and does not colonize epithelial cells [168]. The differential mucus composition in the GI tract of chicken and humans has been suggested to play a role in determining *C*. *jejuni* pathogenicity [169,170]. In vitro studies have shown that chicken intestinal mucins, predominantly those from large intestine, decreased *C. jejuni* binding to human HCT-8 cells [169]. The aforementioned study implied a role for mucin glycosylation in bacterial inhibition, a premise further explored by Struwe et al. [103] who determined that the increased glycan diversity observed in the large intestine corresponded with the high inhibitory effect of mucins from this region on *C*. *jejuni* and lower bacterial density. Studies have shown that *C*. *jejuni* is chemo attracted to pig gastric and bovine gallbladder mucin, L-aspartate, L-cysteine, L-glutamate, and L-serine amino acids, and L-fucose structures [171]. *C. jejuni* can recognize glycan structures such as terminal mannose, NeuAc, galactose and fucose, an interaction that is affected by temperature and oxygen [172]. Furthermore, L-fucose upregulates the *C*. *jejuni* cj0480c-cj0490 genomic island which allows the bacterium to use fucose as a carbon source for growth [173].

## 4. Cow

Unlike pigs and chickens, which are monogastric, bovines are polygastric animals with a four-compartment stomach composed of rumen, reticulum, omasum, and abomasum, followed by the small and large intestines. The abomasal compartment is functionally similar to the stomach of monogastric animals including humans, as it secretes HCl and pepsinogen. In bovines, genome wide analysis has allowed the identification of several secreted (*Muc2*, *Muc5ac*, *Muc5b*, *Muc6*, *Muc7*, and *Muc19*) and membrane bound mucins (*Muc1*, *Muc3a*, *Muc4*, *Muc12*, *Muc13*, *Muc15*, *Muc16*, *Muc20*, and *Muc21*), the majority of which are transcribed in the GI tract of adult healthy cows (Table 1) [104]. Gel-forming mucins are expressed from the abomasum onwards and membrane-associated mucins have a more general distribution throughout the GI tract [104]. In the abomasum are the main secreted mucin genes expressed are *Muc5ac* and *Muc6*, while *Muc2* and *Muc5b* are predominantly expressed in the intestine [104]. Calf small intestinal mucin isolated by density gradient centrifugation has a glycan composition rich in galactose, followed by *N*-acetylglucosamine (GlcNAc), GalNAc, fucose, mannose, and NeuAc [105].

Few studies have characterized the effect of GI infections on mucus composition in cattle, and mostly focus has been given in response to parasitic infections. Nematode infections in the GI tract of cows can affect mucus composition and mucin glycan biosynthesis. This is the case for *C. oncophora* and *O. ostertagi* infections in the small intestine and abomasum respectively (Table 2) [135,136]. Both parasites induce a Th2 like immune response with upregulation of IL-4 and eosinophilia [174]. Infection with the nematode *C. oncophora* results in increased mRNA and protein levels of Muc2 in the small intestine [136]. *O. ostertagi* infection results in thickening of the abomasal mucosa and hyperplasia of mucus secreting cells [135], accompanied by increased transcription levels of *Muc1*, *Muc6*, *Muc20*, and fucosyltrasferases in the abomasum [135]. Despite of *Muc5ac* being commonly found in the abomasum, its expression remained unchanged during infection [135]. Soon after infection of the bovine GI tract, both *C*. *oncophora* and *O*. *ostertagi* induced expression of the mucin glycan biosynthesis enzyme gene *Gcnt3*, in addition to *Gcnt4* and *A4gnt* induced by *O*. *ostertagi* [135,136], further supporting that changes in the mucus niche occur during infection.

Mucins can play a protective role limiting pathogen colonization [158]. Purified bovine Muc1 inhibited binding of *E*. *coli*, *S. enterica*, *Staphylococcus aureus*, and *Bacillus subtilis* to a human intestinal cell line in vitro in a dose dependent manner [157]. Sialic acid, l-fucose, and d-mannose were among the Muc1 carbohydrates involved in both *E*. *coli* and *S*. *enterica* binding inhibition [157]. Enterotoxigenic *E*. *coli* (ETEC) is a common cause of diarrhea in calves [175]. ETEC adheres to calve intestinal mucus through the interaction of bacterial pili and mucus glycoproteins in a pH dependent manner (Table 2) [137]. Different fimbriae recognized different receptors, K99 pili adhered to sialic acid and galactose, while F41 pili bound to desialylated glycans [137]. Unlike ETEC, enterohemorrhagic *E*. *coli* (EHEC) O157:H7 is not pathogenic in cattle. However, the GI of cattle is an important source of this bacterium that can cause disease in humans. Aperce et al. showed that *E*. *coli* O157:H7 grows in the presence of bovine crude intestinal mucus [176] and *E*. *coli* O157:H7 can use mucin carbohydrates found in the cow small intestinal mucus layer as a carbon source for growth [177]. *E. coli* O157:H7 utilizes fucose, galactose, GalNAc, GlcNAc, mannose, and NeuAc as the sole carbon sources when grown on minimal medium, with GlcNAc or sialic acid having the highest growth yield compared to the glucose control [177]. Furthermore, in vitro bacterial competitive assays demonstrated that bovine mucus-derived mannose, GlcNAc, NeuAc, and galactose, give EHEC a growth advantage due to a higher expression of genes involved in carbohydrate catabolism and faster utilization of glycans than commensal bacteria [177]. This phenomenon has also been observed in other organisms, such as the utilization of L-fucose by *C*. *jejuni* carrying the putative fucose permease (*fucP*) gene which is upregulated in the presence of fucose [178]. Similarly, induction of *nanH*, *dctp*, *nanA*, and *nanK* gene expression in *Vibrio cholerae* allows it to utilize sialic acid as a carbon source for growth [179].

## 5. Fish

The fish gastrointestinal tract anatomy differs between species; some have a stomach whereas others do not. Some fish have an intestine with small outpocketings in the anterior region called pyloric caeca to increase the surface area of the digestive epithelium, whereas other species may have a spiral intestine for the same purpose. The Atlantic salmon, *Salmo salar*, a prominent species in aquaculture, has a stomach and pyloric caeca in the anterior part of the intestine [180].

Five orthologues of the human and murine MUC1–MUC20 mucins have been identified in the genome of pufferfish, *Fugu rubripes* [181], and 13 in the zebrafish, *Danio rerio* [182]. Among cultured species, Sveen et al. [107] recently identified seven putative mucin genes of the Muc2 and Muc5 families in Atlantic salmon using annotation, transcription, and domain structure approaches. Pérez-Sánchez et al. [106] identified *I-Muc*, *Muc13*, *Muc18*, *Muc2*, *Muc2*-like and *Muc19* in gilthead sea bream intestine (Table 1). Generally, studies involved in teleost mucin gene analyses rely on homologies with mammalian or zebrafish mucin genes (i.e., a study predating the aforementioned publication by Sveen et al. reported partial mucin sequences with homology to human MUC2 and MUC5 mucins in salmon skin [183]).

There is limited knowledge on fish GI mucin glycosylation (Table 1), except for Atlantic salmon where a total of 91 structures were identified on mucins isolated from pyloric caeca, proximal intestine, and distal intestine using mass spectrometry [51]. The structures present differed between regions: pyloric caecal mucins carried 56 structures, proximal intestine 65 structures, and distal intestine 75 structures [51]. However, within each tissue the glycan profile was remarkably similar; salmon mucin glycans showed much lower inter-individual variation in structure repertoire [51], compared with for example human or pig mucin glycans [52,64]. The major structures found were core 5 and extended core 5, with one NeuAc linked to the reducing end GalNAc in a α2-6 linkage, and most glycans were sialylated (NeuAc, 83–92%), Table 1 [51]. Core 1 and 2 glycans were detected in low abundance [51]. In addition, mucin glycosylation has been studied indirectly in carp by histochemistry and lectin staining of goblet cells [108,109]. β-GlcNAc, α1-6 fucose, α-GalNAc, β-Gal, and sialic acid containing structures were found in common nase, *Chondrostoma nasus*, with higher level of sialyation in the posterior part than in the anterior [109]. In chub, *Squalius cephalus,* the majority of neutral glycans in anterior intestine were fucosylated while β-Gal and β-GalNAc were found in the posterior intestine [109]. Similar to common nase, the highest level of sialylation was found in the posterior intestine [109]. Terminal α-fucose and β-GlcNAc were prominent in the anterior intestine of the Grass carp, *Ctenopharyngodon idella,* while β-Gal and α-GalNAc were more dominant in the posterior intestine, with sialylated and sulfated glycans present in both regions [109]. In the common carp, *Cyprinus carpio*, the major terminal glycan was β-GalNAc; β-GlcNAc was also present, and among acidic structures sulfated glycans were predominant [108,109]. The aforementioned studies show that terminal GalNAc and GlcNAc are often found in fish mucins, and these can be found linked to sialic acids.

The microbiota, infection, mucin expression, and glycosylation appear linked also in fish. Several studies demonstrate mucus related changes during infection (Table2): i.e., the number of mucous cells in the striped trumpeter, *Latris lineata*, increased near the attachment sites of the parasite *Chondracanthus goldsmidi* [184]. *Edwardsiella ictaluri* infection in channel catfish, *Ictalurus punctatus*, altered the expression of genes with similarities to the zebrafish Muc2 and Mucb mucins in the gut [139], and infection with the intestinal parasite *Enteromyxum leei* decreased the mRNA expression of *I-Muc*, *Muc13*, *Muc2* and *Muc2* like mucin genes in gilthead sea bream [106]. An altered mucin glycosylation, such as the appearance of fucose on carp gut mucins after endotoxin treatment, has been suggested to be a microbial clearance mechanism by enhancing bacterial adherence [185], and glycosylation changes that lead to a decrease in sialic acids may give access to cryptic glycan structures that bacteria can utilize [58]. Thirty percent of the studied gut microbiota in turbot, *Scophtalmus maximus,* inhibited the growth of *Vibrio anguillarum*, *A. salmonicida*, and *Aeromonas hydrophila* [186], therefore mucus related changes that affect the microbiota composition has the potential to indirectly affect pathogen density in the gut.

An increased mucus secretion can aid in the removal of pathogens from the epithelial surface [142]. In zebrafish, LPS has been shown to induce mucus secretion using in vivo imaging [187], changes in the intestinal mucin content of common carp occur in response to LPS [185], and *V. cholerae* infection in zebrafish increases the quantity of mucus expelled into the surrounding water [188]. The adhesion capacity and interactions of bacteria in mucus varies with species and host specificity, Table 2. *Vibrio alginolyticus* adheres to crude gilthead sea bream, *Sparus aurata*, skin, gill and intestinal mucus, and is able to use mucus as an energy source [140]. Pathogenic vibrios, namely *V. alginolyticus, V. anguillarum, V. harveyi,* and *V. tubiashi,* are also chemotactic towards skin, gill, and intestinal mucins of gilthead sea bream [189]. Galactose containing epitopes could be of importance for *E. ictaluri* adherence to channel catfish olfactory mucosa, as binding decreased after preincubation with galactose [190]. *A. salmonicida* binds to NeuAc on mucin *O*-glycans, the elimination of which reduces the binding ability of this pathogen to Atlantic salmon mucins [138]. *A. salmonicida* showed higher binding to gut mucins compared to skin mucins, with gut mucins having a higher level of sialylation (mono-, di-, and trisialylated *O*-glycan structures), longer glycan chains, and higher glycan diversity than skin mucins [138]. Thus, it is likely that binding occurs not only to NeuAc, but also rather to glycan structures including NeuAc. Despite being a target for adhesion, *A. salmonicida* is not able to hydrolyze NeuAc from mucins [58]. Atlantic salmon intestinal mucins enhance *A. salmonicida* growth and enzymatic removal of NeuAc increases growth further by exposing GlcNAc [58]. Calcium ion levels and pH also play a role in the adhesion and growth mechanism of *A. salmonicida* in contact with salmon mucins, underlining the importance of environmental factors in fish host-pathogen interactions [58].

## 6. Methodological Considerations for Mucus/Mucin Studies

The interest in mucus and mucins has expanded notably during recent years, and for researchers moving into this field, it is important to be aware of factors that result in commonly used tools to investigate changes after challenge being less suitable for mucin studies compared to other molecules. Firstly, the secreted mucins are very large and do not typically enter standard gels used for sodium dodecyl sulfate–polyacrylamide gel electrophoresis (SDS-PAGE), instead, what is found on such gels are either small (i.e., membrane bound) mucins or fragments of the large mucins, indicating the need for special gels. Secondly, since mucins are to 85% carbohydrate and subject to major post-translational regulation, mucin mRNA levels do not always reflect the amount of mucin/mucus being produced [142]. Histology on its own, without for example metabolic labelling can also be deceptive because important mucus functions are mainly carried out after secretion. Mucin production and secretion rate are important parameters in this context, secreted mucus is lost during processing using most histology methods and mainly the amount of mucus inside the goblet cells is evaluated. A decreased secretion could lead to an increased amount of mucus in the cells, and conversely, an increased secretion could lead to a decreased amount of mucus in the cells, none of which reflects the amount of mucus produced. Furthermore, these methods do not take into account the complexity of mucus, mucins, and mucin glycosylation. Therefore, it is necessary to combine several methods, such as glycan analysis, metabolic labeling of mucins, and histological evaluation to get a more complete understanding of mucus related events.

## 7. Complexities and Potential

Several studies suggest that mucus and mucins are important for regulating growth, composition, and behavior of the microflora (including pathogens), and that these in turn regulate mucus and mucins. For example, the commensal flora can improve the mucus barrier and pathogens can affect mucus via the inflammatory response, thus these factors are intertwined. However, the mucus-pathogen interactions are complex and depend on microbe and host species; therefore, in order to achieve such advancements, more knowledge is needed on this topic. One can envision many aspects where these systems can be utilized to prevent/treat disease, such as via feeds that enhance the mucus barrier and shape a beneficial microflora, potentially decreasing the amount and severity of infections, or via feeding a beneficial microflora directly. Furthermore, breeds may differ in mucus related parameters such as mucin glycosylation, and selective breeding could take mucin mutations and glycosylation into account once an “ideal” mucin repertoire has been identified. There is also potential for temporary “mucus boosting” treatment, for example with the purpose of “flushing out” a pathogen or to prophylactically enhance the barrier to prevent infections from spreading within a site. 

## Figures and Tables

**Table 1:** Gastrointestinal mucin species and glycosylation profile in farmed animals.

Animal	GI Site	Mucin Species	Mucin Glycosylation Profile
Pig	Stomach	Muc5ac, Muc5b, Muc6 [97].	Predominance of core 1 and 2 glycans, terminal Gal, low sialylation and high sulfation [97,98,99].
	Colon	Muc2 and Muc4 [100,101].	Predominance of core 4, and equal distribution of NeuAc and NeuGc [52].
Chicken	Small intestine	Muc2 and Muc13 [102].	Predominance of core 3 and 4 glycans in caecum, small and large intestine. Sialic acid only in large intestine [103].
	Caecum	Unknown	
	Large intestine		
Cow	Rumen	Muc1, Muc20 and Muc16 [104].	Unknown
	Reticulum		
	Omasum		
	Abomasum	Muc1, Muc20, Muc5AC, Muc6 [104].	
	Small intestine	Muc1, Muc20, Muc3A, Muc13, Muc2, Muc5b [104].	Rich in Gal, GlcNAc, GalNAc, fucose, mannose, NeuAc [105].
	Large intestine	Muc1, Muc20, Muc3a, Muc13, Muc2, Muc5b [104].	Unknown
Fish	Intestine	*Sparus Aurata*: I-Muc, Muc13, Muc18, Muc2, Muc2 like, and Muc19 [106]. *Salmo salar*: Muc2.1, Muc2.2, Muc5a.3 [107].	*Salmo salar*: predominance of core 5, extended core 5, and sialylated glycans (NeuAc) [51]. *Cyprinus carpio*: predominance of β-GalNAc and sulfated glycans [108,109]. *Ctenopharyngodon idella*: predominance of terminal fucose and GlcNAc in anterior intestine, and β-Gal and α-GalNAc in posterior intestine [109]. *Chondrostoma nasus*: GlcNAc, fucose, GalNAc, Gal, sialic acid [109].

GI, gastrointestinal; Gal, galactose; NeuAc, *N*-acetyl-neuraminic acid; NeuGc, *N*-glycolylneuraminic acid; GlcNAc, *N*-acetylglucosamine; GalNAc, *N*-acetylgalactosamine.

**Table 2:** Mucin binding pathogens and mucin response to infection in the gastrointestinal tract of farmed animals.

Animal	GI Site	Pathogen Binding	Mucus/Mucins Binding Structures	Mucin Response to Infection
Pig	Stomach	*Helicobacter suis*	Galβ3GlcNAcβ3Galβ4Glcβ1, charged structures, pig gastric mucins, and glycolipids [97].	Unknown
	Small intestine	*Escherichia coli K99*	Sialic acid on mucins and glycolipids [129,130,131].	
	Colon	*Brachypira hyodysenteriae*	Pig colon mucins [101].	Increased Muc2 and Muc5ac expression, and decreased Muc4 expression [100,101].
		*Brachyspira hampsonii*	Unknown	Decreased Muc4 expression [100].
		*Salmonella Typhimurium*		Muc5ac expression [132].
		*Trichuris suis*		Muc5ac expression [133].
		*Trichinella spiralis*		Mucin increase [134].
Chicken	Small intestine	*Clostridium perfringens*	Unknown	Increased Muc5ac expression, decrease in Muc2 and Muc3 expression [102].
Cow	Abomasum	*Ostertagia ostertagi*	Unknown	Increased expression of Muc1, Muc6, Muc20 [135].
	Small intestine	*Cooperia oncophora*		Increased expression of Muc2 [136].
		*Escherichia coli* K99	Sialic acid and galactose [137].	Unknown
		*Escherichia coli* F41	Desialylated glycans [137].	
Fish	Intestine	*Aeromonas salmonicida*	NeuAc on mucins of *Salmo salar* [138].	Unknown
		*Edwardsiella ictaluri*	Unknown	Altered expression of genes similar to zebrafish Muc2 and Muc5b in *Ictalurus punctatus* [139].
		*Enteromyxum leei*		Decreased mRNA expression of I-Muc, Muc13, Muc2, Muc2 like mucin gene in *Sparus aurata* [106].
		*Vibrio alginolyticus*	Intestinal mucus of *Sparus aurata* [140].	Unknown

GI, gastrointestinal; NeuAc, *N*-acetylneuraminic acid.
